# Ultra-Processed Food Intake and Cognitive Impairment in Nationally Representative Older U.S. Adults

**DOI:** 10.1093/geroni/igaf122.2913

**Published:** 2025-12-31

**Authors:** Heejin Lee, Elizabeth Ludwig-Borycz, Cindy Leung

**Affiliations:** Harvard School of Public Health, Boston, Massachusetts, United States; University of Michigan, Ann Arbor, Michigan, United States; Harvard T.H. Chan School of Public Health, Boston, Massachusetts, United States

## Abstract

Ultra-processed food (UFP) consumption accounts for more than 50% of energy intake in the US, steadily increasing among older adults over the decade. UPF consumption is associated with cognitive decline, but fewer studies have examined the association between UPF consumption and individual cognitive domains. In this study, we examined associations between UPF consumption and impairment in memory, executive function, language, visuospatial, and orientation in the nationally representative Health and Retirement Study (HRS) of adults aged ≥50. Participants without dementia or memory problems at baseline (2012 HRS) were included (n = 1,408). Dietary intake was assessed using the food frequency questionnaire from 2013 Health Care and Nutrition Study (HCNS). UPF was classified using the NOVA categorization. The percentage of energy intake from UPF was grouped into sex-specific quintiles (Q). Cognitive impairment was assessed from the 2016 Harmonized Cognitive Assessment Protocol (HCAP) based on cognitive domain scores, defined as > 1.5 SDs below the mean or a T-score=35. Weighted multivariate-logistic regression was used to estimate the associations between UPF consumption and cognitive outcomes. In the analytic sample, median UPF intake was 42.3% energy/day. After adjustment for baseline sociodemographic and health characteristics, higher UPF consumption was associated with greater impairment in executive function (Q4 vs Q1, OR 2.12, 95% CI 1.17-3.83; Q5 vs Q1, OR 1.77 95% CI 0.98-3.17; P-trend=0.04). UPF consumption was not associated with impairment in other cognitive domains in multivariate models. Our findings highlight the need for support in reducing UPF intake for better cognitive health among older adults.

